# The dental demolition derby: bruxism and its impact - part 1: background

**DOI:** 10.1038/s41415-022-4143-8

**Published:** 2022-04-22

**Authors:** Mark L. T. Thayer, Rahat Ali

**Affiliations:** 41415113409001 Consultant and Honorary Lecturer in Oral Surgery, Liverpool University Dental Hospital, Pembroke Place, Liverpool, L3 5PS, UK; 41415113409002 Consultant in Restorative Dentistry, Liverpool University Dental Hospital, Pembroke Place, Liverpool, L3 5PS, UK

## Abstract

Bruxism may lead to changes or damage to the oral and perioral tissues. Bruxism may occur during sleep or when awake. Many patients will not require active management; however, for some, intervention is required. Control of bruxism may be difficult, if not impossible, but the need exists for preservation of the dentition and quality of life. A prediction of risk to the tissues for the planning of interventions is difficult and relies upon evidence of past damage and assessment of future risks. Treatment options may need to be imaginative and rescuable. This series of papers will review the aetiology of bruxism, its impacts and treatment strategies for persistent bruxers who are at risk of, or suffering, tissue damage.

## Key points

- Bruxism is not just simply grinding of the teeth and is more than temporomandibular disorder.
- Bruxism is a centrally driven sleep disorder that often has a familial and long patient history.
- Bruxism is initiated and/or exacerbated by selective serotonin reuptake inhibitors.
- Bruxism can be very destructive to the dental and oral tissues and leads to quality of life issues. Decisions as to when to intervene are difficult and an intervention needs assessment is introduced.

## Introduction

**Fig. 1:**
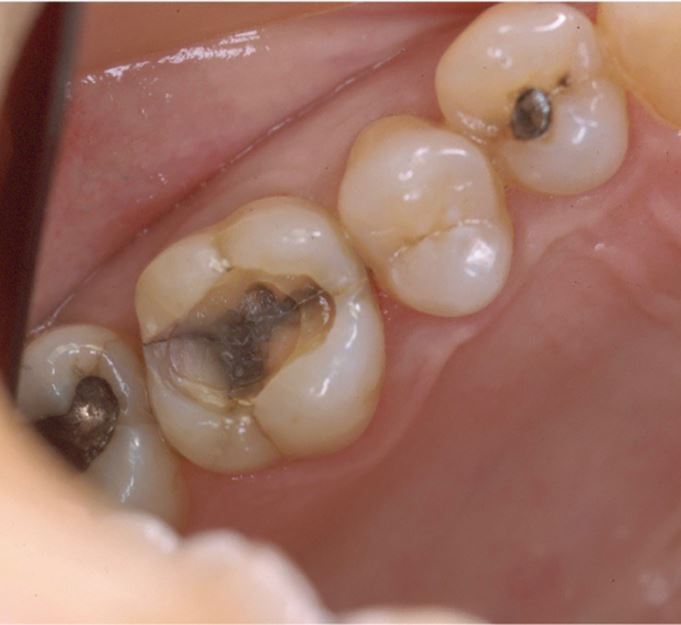
Fractured distopalatal cusp in bruxist

**Fig. 2:**
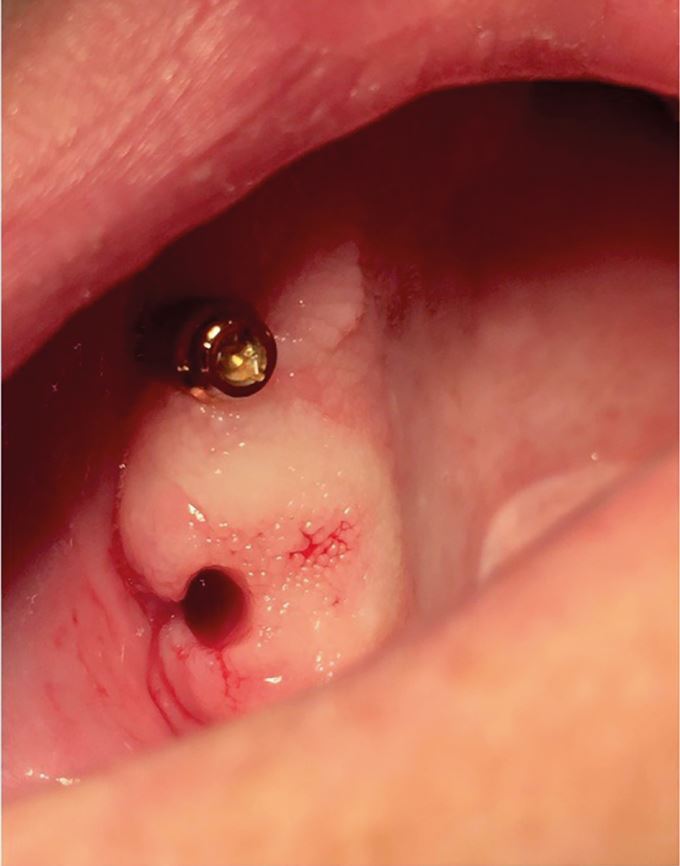
Fractured implant fixture in bruxist

**Fig. 3:**
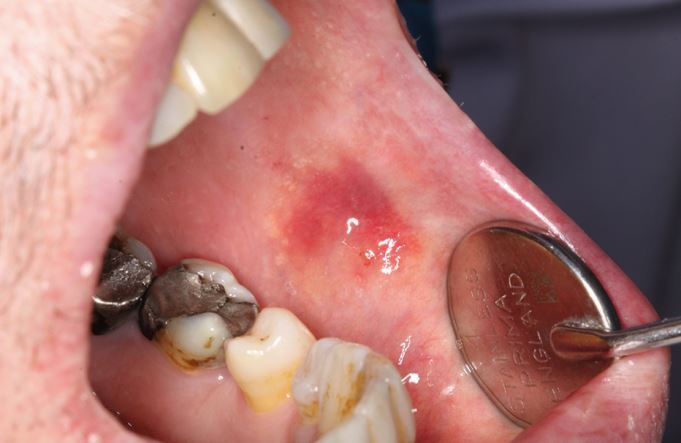
Soft tissue trauma related to nocturnal bruxism

**Fig. 4:**
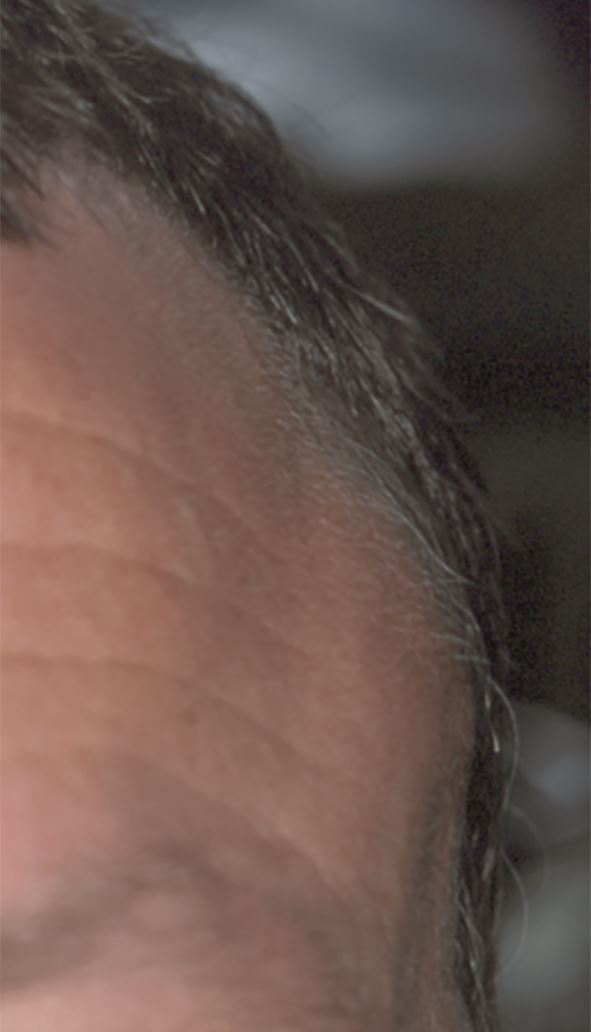
Hypertrophy of left temporalis muscle related to bruxism

Bruxism was defined in 2013 as 'repetitive jaw-muscle activity characterised by clenching or grinding of the teeth and/or by bracing or thrusting of the mandible' by international concensus,^1^ with supplemental classification of sleep or awake bruxism. It has also been classified as a sleep movement disorder.^2^^,^^3^ Tooth surface loss (TSL) is a well-recognised phenomenon, evident in all age groups, with well-defined management strategies.^4^^,^^5^^,^^6^^,^^7^ TSL is multifactorial in nature but one aspect leading to TSL is parafunctional behaviours of the oral and perioral tissues, in particular, bruxism. Damage from bruxism can be minor or substantial, affecting hard tissues, with attrition of occlusal surfaces of the teeth, fractures of teeth or restorations (Fig. 1), including implant retained restorations (Fig. 2) and can lead to damage or changes in the soft tissues via soft tissue trauma (Fig. 3), ulceration, hypertrophy or hyperplasia (Fig. 4). There have even been reports of parotid duct obstruction with consequent symptoms.^8^^,^^9^ Function may be impacted and aesthetics changed, leading to negative psychological impact and in some, social isolation and poor quality of life.^10^^,^^11^^,^^12^ Persistent increased muscular activity may become symptomatic, with facial pain and aspects of temporomandibular symptoms, but also may be symptomless. The impact of the activity can damage tissues that may then become painful, for example with a fracture of a tooth, but often with no apparent pre-existing history of bruxism. Control of bruxism and other parafunctional activity and the damage that these can produce may significantly improve function and quality of life, but in addition, may also improve outcomes for patients with reduced clinical time and reduced financial burden, to both the patient and the health service. A primary issue with bruxists is predicting the long-term outcomes. While many will not require active intervention, others do require methods of protection of the dentition and tissues. Risk assessments for prescribing are difficult and rely on historical evidence of tissue damage and ongoing behaviour patterns, with real-time predictors of future risks.

## Epidemiology

While increased muscular activity may often be symptom free, it is common. Incidence of bruxism is reported as between 5-30%,^13^^,^^14^ with prevalence appearing similar across the world. Bruxing may occur at night (sleep bruxism), during the day (awake bruxism) or both. There is no sex predilection, but incidence decreases with age, with the peak incidence lying in adolescence and younger adulthood, with a slow and linear decline^13^ in incidence over 40 years of age to a rate of about 3% in older patients (greater than 60 years).^2^^,^^13^^,^^14^ There is an association, although limited, with stress, anxiety and poor mental health and a number of other environmental, extrinsic factors.

## Is it bruxism?

Or when is bruxism not bruxism? 'Bruxism' can be a catchall term used by many to describe grinding or clenching of the teeth, primarily at night. Despite the above definition, it is important to draw a distinction between 'just' increased functional activity and 'real' bruxism. Rhythmic masticatory muscle activity (RMMA) occurs during sleep but appears to be a normal component of sleep behaviour activity for many. Much of the literature looks at sleep bruxism and there is little considering awake bruxism. Lavigne and colleagues^15^ studied both (sleep) bruxers and non-bruxers, finding that the non-bruxist patients also demonstrated this RMMA during sleep, but, as might be expected, there were differences - the bruxing patients showed more episodes of RMMA, with increased bursts per episode and bursts of a greater amplitude yet at a shorter duration. This may be an important finding, as it would appear that bruxing can substantially increase the force applied to teeth.^16^^,^^17^^,^^18^ It seems reasonable to conclude that the cumulative force applied to the teeth is the difference between the two similar activities and leads to the potentially destructive impact of sleep bruxism. Others^1^^,^^19^ have suggested that in excess of four episodes of increased muscle activity and grinding per hour is a breakeven point above which damage can occur to tissues. The difficulty is identifying one from the other and predicting how sleep or awake bruxism will impact in the long-term. This distinction appears repeatedly through the literature but with little clarity in deciding on the defining characteristics of (destructive) bruxism from RMMA, despite the real need for this to aid planning and avoid unnecessary damage to tissues and also to avoid unnecessary treatment. In an expansion of the definition paper, Manfredini and co-authors^20^ explore the concept of bruxism as a continuity from 'simple' nocturnal muscular activity to destructive bruxism, acknowledging the potential complexity of determining where a patient may lie on the spectrum. The title of the paper tells it all: 'From cut-off points to a continuum spectrum'. The concept that it is possible to identify a limit beyond which bruxism is destructive is probably inherently flawed, as it is different for every patient.

It is also important to draw a distinction between temporomandibular disorders (TMDs) and bruxism. TMDs are best considered as a biopsychosocial phenomenon, with patients responding to a range of factors initiating and maintaining parafunctional activity, with consequent symptomology of pain or joint issues. Painful TMDs are not the same as bruxism, nor RMMA; TMDs are typically responsive to environmental factors changing behaviour and there is little solid evidence that pain in TMDs is linked with 'true' bruxism (awake or sleep). While there would appear to be a number of genetic risk factors for painful TMD, the genetic factors are broadly different to those for sleep bruxism.^21^

Diagnosis of bruxism is typically completed via questionnaire, symptom history and examination, with quantifying assessments of bruxism done by the use of electromyography (EMG) and polysomnography (PSG)^1^. In reality, these do not actually tell the clinician if the patient is bruxing, only that the muscles are operating when not expected at night and that the teeth may be in contact. The activity pattern may infer bruxism, for example, the international research criteria for (sleep) bruxism^1^^,^^20^ is the following:

- Four or more episodes of increased activity per hour
- More than 6 bursts of activity per episode and/or 25 activity bursts per hour of sleep
- At least two episodes of grinding sound in a research environment.

There is, however, a lack of validity of these definitions as there is no actual solid evidence that these are correct. A method of recording intercuspal pressure might assist in the more accurate determination of what is, or is not, bruxism. However, the reality is that the use of EMG, PSG and bite force measures is not practical outside the sleep laboratory. As the ability of the researcher and clinician to gauge a specific point when bruxism becomes a 'problem' is so vague, the clinician will inevitably need to rely upon careful history and comprehensive examination, with real time monitoring and understanding of the behaviour of the tissues, coupled with the astute application of clinical judgement.

It is interesting to note that some papers consider generic bruxism as a risk factor for other issues and tissue damage. That also means that patients who brux do not necessarily produce tissue damage or tooth wear, as outlined above. For this paper series, the authors will consider bruxism, whether sleep or awake, as a given and that it is leading to tissue damage. Despite the assertion above that identifying a cut-off point for determining when bruxism is destructive is probably not possible, the authors will consider the 'when you really need to intervene' and the 'how to intervene', in what they hope is a rational format.

## Aetiology

The aetiology of bruxism is considered a multifactorial, but centrally driven, process. This will be reviewed under aspects that can predispose, initiate and promote and those that maintain bruxing (Table 1).

**Table 1:** Bruxism risk factors

Predisposing factors	Initiating factors	Perpetuating factors
Genetic predisposition	-	Genetic predisposition
Childhood history of bruxing	Trauma	Psychological stress
Family history of bruxing	Psychological stress	Mental health issues
Learning disabilities	Mental health issues	SSRIs
Parkinson's and other movement disorders	Specific (recent) occlusal interference	SSRIs/SNRIs
Genetic inherited disorders (eg Prader-Willi syndrome)	SSRIs	-
Psychological stress	SSRIs/SNRIs	-
Mental health issues	-	-
GORD	GORD	GORD
Key: GORD = gastro-oesophageal reflux disorder; SSRI = selective serotonin reuptake inhibitor, SNRI = serotonin and norepinephrine reuptake inhibitor.		

### Predisposing factors

Predisposing factors are risk factors that may make a patient more likely to initiate bruxing activity. These may be intrinsic or extrinsic.

### Intrinsic factors

Discussions with patients frequently (but not exclusively) reveal a long-standing history of teeth clenching or grinding. A history of childhood grinding at night is common and a familial history is also frequent. These histories fit with assessment of bruxism as a sleep movement disorder. Research into potential genetic drivers to bruxism suggests that there are both genetic and epigenetic aspects leading to an individual who is at risk of developing bruxism, in particular, sleep bruxism^22^ and that bruxism is likely to be centrally driven rather than a result of peripheral influences, such as from the occlusion. Research has shown that several genes relating to dopamine biology appear to be associated with bruxism in children.^23^^,^^24^^,^^25^ Age leads to reduced dopamine receptors in the brain, which would align with reduced bruxism with age.

Also, 5-hydroxytryptamine (serotonin) metabolism appears to play a role in the central drive^24^^,^^26^^,^^27^ and selective 5-hydroxytryptamine reuptake inhibitors have been identified as being implicated in bruxism^28^^,^^29^ in adults.

Bruxism may also present as a facet of other nervous system disorders, such as Parkinsonian disorders, cerebral palsy, or other movement disorders.^30^

Sleep disorders may pose an intrinsic risk for bruxism. The majority of sleep bruxism episodes appear to occur during non-rapid eye movement and light sleep at periods of microarousal from sleep; bruxing appears in episodes around the period of arousal. Such episodes are frequent (8-15 times an hour) but last only a short time (3-10 seconds).^2^ Obstructive sleep apnoea may seem a logical culprit, but the association with bruxing is limited and in fact, the association with snoring is greater.^31^ It is proposed that sleep bruxism has a protective effect by moving the mandible when hypopnoea occurs, which would be a centrally driven arousal process.^2^^,^^32^ It is also postulated that bruxing activity can produce lubrication of the oropharyngeal tissues by inducing salivary flow and is associated with swallowing activity in a substantial proportion of cases.^33^

### Extrinsic factors

The primary extrinsic risk factor predisposing to bruxism appears to be psychological - stress, anxiety and depression. This appears to be more strongly associated with daytime awake bruxism than sleep bruxism, which would make sense given the likely genetic and central nature of sleep bruxism. Literature^2^^,^^34^^,^^35^^,^^36^^,^^37^^,^^38^^,^^39^ on this, however, is mixed and the associations not completely clear. One confounding factor in this association is the need for antidepressant therapy for patients with mental health issues, as some of these agents appear to be linked with bruxism. This is explored below. Other predisposing factors include smoking (which links with obstructive sleep apnoea), medication and recreational drug use. Alcohol use appears to have relatively limited association.^31^

### Initiating factors

In most cases, it is difficult to pin down a specific initiation, especially when there is a long history of bruxism; however, there is a range of risks that may lead to initiation of bruxism. Perhaps primary among these are mental health issues. There is a clear association between mental health issues and awake bruxism,^34^^,^^35^^,^^36^^,^^37^^,^^38^^,^^39^ with a significant increased risk of awake bruxism occurring during episodes of stress or anxiety. The relationship with sleep bruxism is less clear cut. Mental health disorders such as schizophrenia are also implicated^40^^,^^41^ but those such as the personality disorders seem less commonly associated. Eating disorders do not seem to specifically increase the incidence of bruxism but are rarely mentioned in bruxism literature.

There is increasingly good evidence of a link with selective 5-hydroxytryptamine reuptake inhibitor (selective serotonin reuptake inhibitor [SSRI]) use,^42^ especially citalopram and sertraline, which may be dose linked, as well as selective 5-hydroxytryptamine and noradrenaline reuptake inhibitors (serotonin and norepinephrine reuptake inhibitors [SNRIs]) such as venlafaxine, which appears strongly linked with bruxism. Kara and colleagues^43^ demonstrated the onset or exacerbation of sleep bruxism in patients commencing SSRI therapy. The onset appears fairly rapid - within days. Here again, the mechanism appears to be related to dopamine activity and its disturbance by SSRIs. Dopamine inhibits spontaneous masticatory muscle activity and 5-hydroxytryptamine interferes with this action. Loss of this inhibition may therefore disinhibit muscular control and lead to the initiation of bruxism.^44^ While their study looked at sleep bruxism specifically, it is impossible to escape the view that this may operate in awake bruxism as well.

However, in general, the potential for changed function is linked to a change in muscle tone as a response to mental distress. As far back as the late 1980s, Rugh^45^ (quoted by Oekson)^46^ demonstrated increased muscle activity linked to stress, with clear linkage between stressful life episodes followed by sharp increases in muscular activity. Parallel evidence shows that mental health issues significantly increase the risk of symptoms of TMDs.^47^ Patients presenting with TMD largely show increased activity in the muscles of mastication, with bruxism frequently blamed for the development of painful TMD. Bruxism may be a risk factor for TMD^48^ but is not necessarily the cause of painful TMD. However, overall, it seems likely that the primary initiating factor for a proportion of cases (mainly awake bruxism) lies within the psychological axis of the condition.

In a few unusual cases, a trigger can be identified, such as trauma (for example, a car accident and associated whiplash). Neuroimaging has identified the involvement of parts of the brain associated with the hypothalamic-pituitary-adrenal axis (HPA) in bruxism and also implicated in TMD and post-traumatic stress disorder (PTSD).^49^^,^^50^ Disturbances in the HPA are significantly implicated in anxiety. Here, it is suggested that bruxism, PTSD, obsessive compulsive disorder and other stress-related psychiatric disorders are due to a dysfunction of this system and it is suggested that a negative feedback loop exists which may be important in the initiation or regulation of bruxing.^49^^,^^51^

**Fig. 5:**
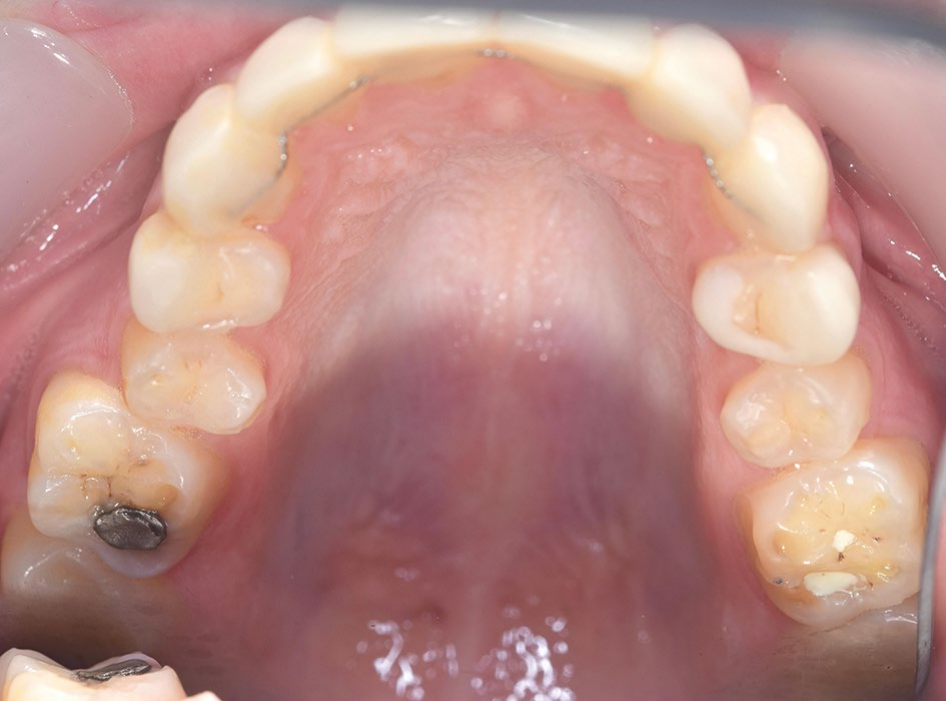
Enhanced erosion evident in a 21-year-old with bruxism and GORD

**Fig. 6:**
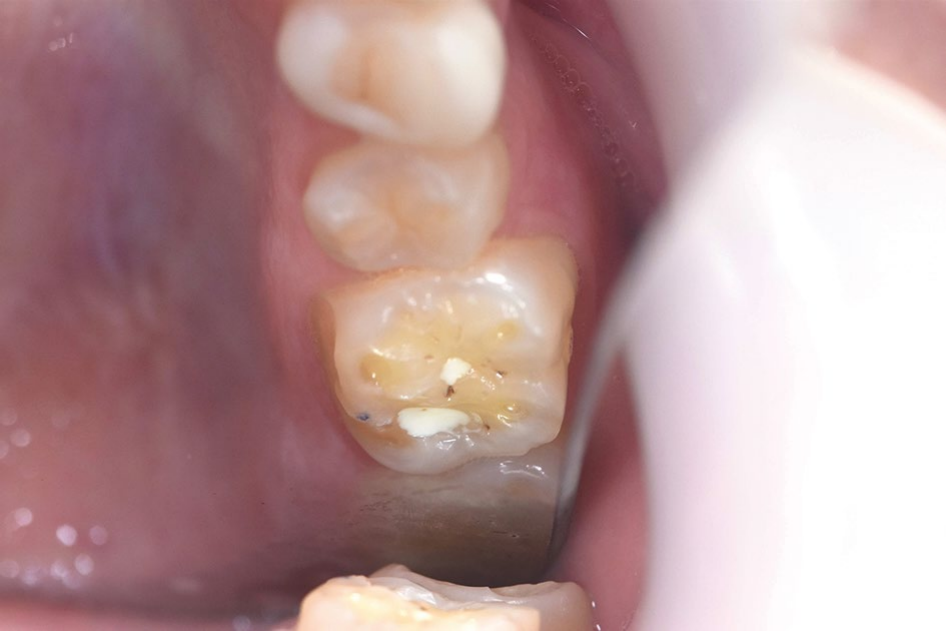
Close-up of 26 in patient from Figure 5. Note the wear faceting across cusps as well as clear evidence of palatal erosion. Treatment need stratification score: 23

A number of studies have shown a strong association between gastro-oesophageal reflux disorder (GORD)^31^^,^^52^^,^^53^ and bruxism. In experimental subjects without GORD, acidification of the stomach initiates bruxism. At first sight, this might seem an odd association and a direct cause and effect is not clear. However, it is proposed that the link may be related to microarousal episodes of sleep disturbance that then initiate bruxism, possibly to lubricate the oropharynx. Disturbed sleep is a common facet of GORD. There are confounding factors, such as the effects of stress on both bruxism and GORD; however, the work by Ohmure and colleagues^54^ clearly shows the initiation of bruxism in non-bruxists by acidification of the stomach. GORD is also an intrinsic risk factor for tooth surface loss that may then be accelerated by bruxism. The dental implications of GORD and acidification of the oral environment are well documented. Figure 5 shows a case of tooth surface loss in a very young bruxist (21 years old) with gastric reflux and significant mental health issues. There is obvious erosion of the teeth but there is also clear attrition with wear facets across cusps of the molars (Fig. 6). This patient would warrant early intervention to control tissue damage.

The evidence therefore suggests that bruxism is a centrally mediated phenomenon that has multiple possible routes and mechanisms for initiation.

### Promoting and maintaining factors

There is relatively little literature looking at reasons that bruxing continues or is exacerbated. However, some aspects of the predisposing and initiating factors will also come into play.

## Does bruxing actually matter? The impact of bruxism

Opinions on the importance or impact of bruxism vary. In their paper in 2018, Beddis *et al.*^55^ suggested that sleep bruxism did not require intervention in most cases but acknowledged that some patients will require intervention due to tissue damage. Yet, as noted, bruxism can lead to a range of issues that can impact on quality of life. In assessing need, it is important to consider the patient's presenting complaint and the historical evidence of damage from bruxism. However, it is clear that simple assessment of tooth wear (as opposed to tooth surface loss) *per se,* is a poor indicator of risk to the patient from bruxism of any origin.

When considering the impact of bruxism, whether awake or asleep, both hard and soft tissues should be considered. While extreme attrition of the dentition is very clear, changes within soft tissues may be more subtle. However, most changes are relatively easy to identify and flag during routine examination and a detailed examination may then be instigated to assess the risk posed to the patient's oral health. Issues to review are shown in Table 2.

**Table 2:** Problems associated with bruxing-induced damage to oral tissues

Hard tissues	Soft tissues
Pain	Pain
Coronal fractures	Recurrent trauma
Tooth loss	Muscle hypertrophy
Attrition + / - pulp exposure	Pulp exposure
Abfraction lesions	-
Acceleration of erosive damage	-
Recurrent restoration failure	Parotid obstruction
Cosmetic impact	Cosmetic impact
Psychological impact	

The psychological impact varies tremendously. Some patients are remarkably sanguine and accept dramatic tooth (tissue) loss over time as just part of life, while others worry to an obsessive amount about the most minor loss of enamel. Discussions with patients who have significant worries in the presence of relatively minor changes often reveals an increased level of general (or trait) anxiety, mental health issues in general and stressed personal circumstances. The impact on cosmetics and the patient interaction with friends and family can be a very strong driver to seek correction of tooth surface loss, even when such correction is not warranted. In very crude stratification, older patients tend to be pragmatic, while younger patients tend to have a very low threshold for concern. This is an issue, as these significant levels of anxiety impact on the patient's quality of life and may lead to inappropriate demands for treatment.

Risk assessment is complex, involves a large amount of clinical judgement and can be fraught with potential pitfalls. A clear diagnosis of bruxism is required and this is well covered by Beddis *et al.,*^55^ although this looked specifically at sleep bruxism. The criteria for a diagnosis of sleep bruxism is noted previously and relies upon self-reported information with clinical signs, but the gold standard is polysomnography. This does, however, only address the sleep phase of bruxism and is not realistic for day-to-day dental practice. Diagnosis of awake bruxism must rely much more heavily on patient reports, behavioural analysis (for example, habits and daytime activities) and clinical signs.

Treatment need is driven by both the extent of tissue damage, the patient expectations and the modifying factors. A potential need stratification matrix is shown in Table 3, which may offer a method to enhance and rationalise the decision-making process, increasing decision defensibility. This assessment matrix has been trialled by the authors, both prospectively and retrospectively and appears to provide an accurate support to the assessment process. The clinical assessment allows completion of the matrix, with an overall assessment of low, medium, or high potential need for intervention.

**Table 3:** Tooth wear and bruxism intervention need stratification

Risk factor	Score 1	Score 2	Score 3	Outcome
Age	>40	-	<40	-
Bruxing history	N	-	Long-standing Childhood	-
Present bruxing	Infrequent	-	Common	-
Disturbed sleep patterns	N	-	Y	-
Extent of attrition/TSL	Low	Medium	Extensive	-
Fractures of teeth/restorations	N	-	Y	-
Number of fractures of posterior teeth/restorations (only score if above = Y)	<3	-	>3	-
Soft tissue changes/injury	N	-	Y	-
GORD	N	-	Y	-
Psychological status	Low impact	-	High impact	-
SSRI/SNRI use	N	-	Y	-
Dietary influences	N	-	Acid-rich diet	-
Smoking	N	-	Y	-
**Total**				
13-17	Low need	-	-	-
18-21	Medium need	-	-	-
>22	High need	-	-	-

## Why an intervention needs assessment?

The issue facing patients is the potential of delays in intervention, until such time as the dentition is extensively damaged, requiring extensive reconstruction and with an impaired long-term prognosis for the dentition. While extensive damage is easy to identify, early identification of patients who are at significant risk of extensive tooth wear is less clear, yet more crucial for prevention of damage. The matrix is described as an intervention needs matrix, rather than a risk assessment matrix, as by definition, all patients in this group are risk patients.

## Designing the intervention needs matrix

Design of a matrix requires assumptions and review to refine. Some aspects may seem speculative but have a logical design rationale. For example, the number of fractured teeth; three as a watershed might appear to be simple guesswork. However, it should be considered that the fracture of 3 (posterior) teeth represents approximately 20% of the functional posterior units - more if orthodontics has led to early premolar loss. Anterior teeth appear to show substantial fracture less commonly and small amounts of enamel chipping are not included as there are other confounding reasons for incisal edge fracture, not least external trauma. Multiple crowns of posterior teeth should be assumed to be likely to represent at least one fracture (more if confirmed with patient records or clear history) but may be ignored in patients presenting with a high caries rate as the caries is more likely to be the cause of extensive damage.

Soft tissue changes relate to muscular hypertrophy and other significant changes, but not those such as linea alba or tongue scalloping, as these present in many patients with non-destructive habits and 'simple' TMDs.

Each area is assigned a score, depending on the significance of the issue identified. For example, patients over 40 score low, as tooth wear will be more in line with age. A score of 13-17 equates to a low potential need, 18-21 as medium and greater than 22 as high. The scoring will not allow a score below 13 as this loses sensitivity; there is a need for dilution of some findings to avoid a situation where everyone scores in the 'treatment needed' group.

**Fig. 7:**
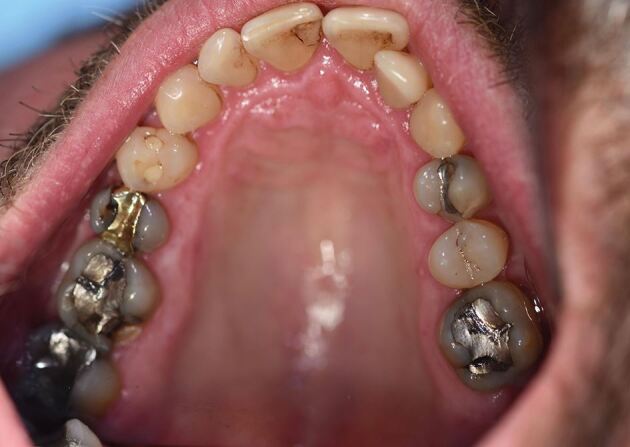
Rhythmic masticatory muscle activity. Note there is only one cusp fracture and minimal wear faceting. Treatment need stratification score: 14

**Fig. 8:**
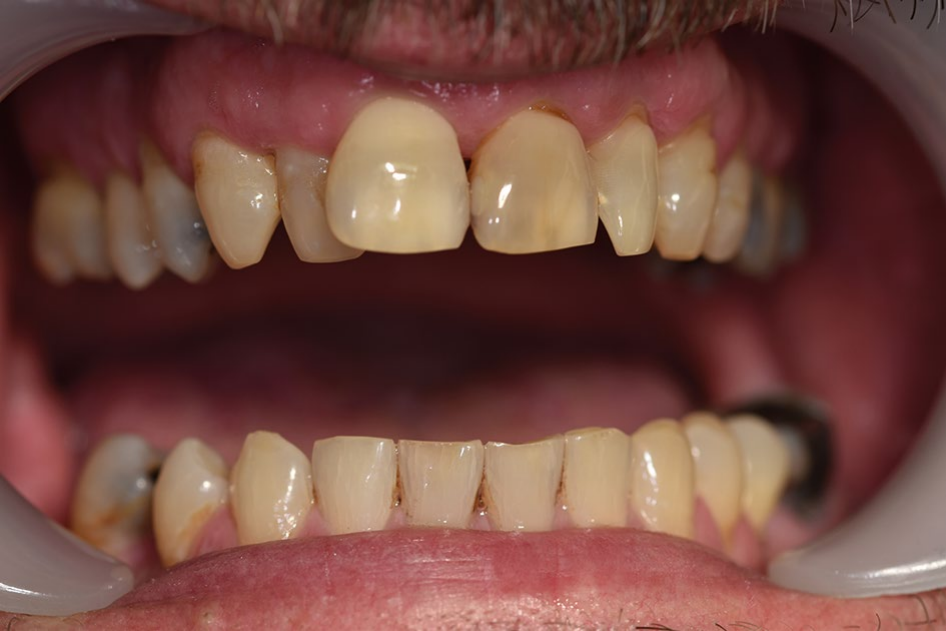
Patient from Figure 7 - 45-year-old with rhythmic masticatory muscle activity. Note that tooth wear is in line with normal physiological wear

This material can then form the basis for discussions with patients as to the appropriateness of intervention (not the type of intervention). What these assessments cannot establish is a cut-off point beyond which treatment is positively indicated, nor when it is positively contra-indicated, which is in line with the earlier discussion in relation to the difference between cut-off points and the continuum of bruxism. In reality, this matrix looks at 'if' the patient will need intervention, rather than 'when'*.* Figures 7 and 8 show a 46-year-old patient with RMMA scoring 14 and when compared to Figures 5 and 6 (score 23), shows this variation of need in action.

Application of this matrix will be discussed in context in the next papers of the series.

## Conclusion

Bruxism is much more than simply grinding of the teeth. Sleep bruxism is a different condition to awake bruxism, with different and multifactorial causation and risk factors. Sleep bruxism appears to be a sleep disorder, whereas awake bruxism is probably more associated with psychological stress and medication. In both cases, the clinician needs to deploy diagnostic skills with discretion and intelligence and must be aware of the potential damage that bruxism can cause to the oral tissues.
